# Commentaries on *the Organon*

**Published:** 1892-05

**Authors:** B. Fincke

**Affiliations:** Brooklyn, N. Y.


### COMMENTARIES ON THE *ORGANON.*

*The Universality of the Homœopathic Natural Law of Healing.—Organon,* §§ 16, 25, 26, 29, 64, 111, 112, 186.

B. FINCKE, M. D., BROOKLYN, N. Y.*

The assumption of universality of the homœopathic natural law of healing for other modes of healing than that by administration of medicine was declared at our last meeting to be unwarranted, and was stigmatized as fanaticism. The discussion

---

* Read before the New York Homœopathic Union.

following was by no means exhaustive, though very interesting, but the general opinion of the debaters seemed to incline the other way. Since, also, the half-way men in our profession have taken up the position of the objectors, and on the ground of the insufficiency of the law, admit in their practice measures directly opposite to its teachings, it is of the greatest importance, if possible, to settle this question, and for this reason I crave your indulgence in presenting some remarks as my mite to its solution.

In going to the fountain-head of our homœopathic knowledge, the *Organon*, we find in § 25: that "*Pure experience teaches that that medicine which, in its action upon healthy human bodies has proved itself able to generate the greatest number of symptoms in simility to those to be found in the case of disease which is to be healed, does also in duly potentiated and lessened doses, speedily, thoroughly, and lastingly cancel the totality of the symptoms of this state of disease.*" "*This,*" § 26 continues, "*rests upon that homœopathic natural law lying at the foundation of true healing: a weaker dynamic affection in the living organism is extinguished lastingly by a stronger one if this (differing from it in kind) is very similar to that in its utterance.*" The examples for this statement in the note refer to the action of all kinds of agencies, not medicinal alone, upon the living organism. They are taken from natural philosophy, from public hygiene, from the discipline of armies, from the stratagems of war, from the sympathy of fellow-men, from great political events. Hahnemann could not have given such widely differing examples of the action of external agencies upon the organism if he had not intended to show that the homœopathic natural law to which he pointed was of a general kind, covering not only the medicinal administration to the sick, but also various other efficient methods which may have a healing effect if that organism is by some cause or other diseased.

It is true that a natural law is not necessarily a universal law, because it is a law of nature, and nature is covering the universe. The law of reflection of light, *e. c.*, is a law governing only the propagation of light in the universe; it is not universal, because it does not apply to anything but to light, and there are other things than light in the universe to which it does not apply. To be universal a law must find its application in anything that moves, acts, and exists. A universal law makes out of the universe a whole, a totality of all phenomena that the human mind can conceive similarly, as we discover in the totality of the symptoms what we can conceive of the disease distuning the life-force. If, therefore, Hahnemann had only spoken of a natural law, those who consider the homœopathic law only as a specialty of healing, insinuating that there may be other special laws of healing which might upset and supersede that law which he propounded, would have in him a defender of their opinions. But it is not so. Hahnemann detested this kind of reasoning. There was no uncertainty about this matter with him. In determining that there was a natural law lying at the foundation of healing, he clearly gave us to understand that it was not merely a natural law, but a *homœopathic* natural law. From the examples given it appears that he meant a deep original principle which, as it has its sway in the whole world, governs, also, the science and art of healing. Though he did not express and formulate it, we see from numerous sections in the *Organon* what he means. In § 111 he says: "*That medicinal substances in their morbid change of the sound human body act according to certain eternal laws of nature.*" In § 64 he distinctly maintains that to the action of the remedy the reaction of the life-force follows. In § 112 he also speaks of the action of the remedy and reaction of the life-force. Though in his view on the action and reaction the clearness of the perception is marred by confounding the indication by simility with the action of the remedy, yet the idea of action and reaction in healing is clearly perceptible. In § 16 he maintains that the distunement (*verstimmung*) of the organism by dynamic causes can only be removed by similar dynamic remedies. Now, if the remedies act on the life-force, it is clearly action and reaction. In § 29 he explains healing by substituting a similar artificial disease-potency to the natural disease-potency on account of the homœopathicity of the first which gives it the power to equalize the opposing disease-potency, so that the life-force is enabled to resume its normal course. Here again is action and reaction clearly discernible.

Three years after the last edition of the *Organon*, in the preface to the fourth volume of *Chronic Diseases*, he labors at an explanation, which, though not an improvement upon his view previously expressed in the *Organon*, yet points again to the same action of the remedy and reaction of the life-force. It is singular, indeed, that Hahnemann did not, for the homœopathic natural law of which he speaks in § 26, fall back upon that great law of equality and contrariety of action and reaction which covers all motion. Since there is no absolute rest (no Nirvana) but only relative rest—for as far as can be perceived the whole universe is constantly in motion, as a whole as in its parts down to the infinitesimals, this law is most assuredly a universal law. It is essentially the law of healing without which a scientific conception is impossible. This law accounts as well for the simplest mechanical motion as for the most intricate organic processes of sickening and healing, which are the most elaborate motions known. Just as the force in action calls out the reaction in the body opposed to it, and equalizes its resistance in virtue of its similar dynamic quality, so the healing force in virtue of its simility of dynamism overcomes the similar sickening force in the living organism according to this universal law. From all this it appears, that the adoption of this universal law of motion, that action and reaction are equal and contrary, for that *homœopathic* natural law of which Hahnemann speaks, is very desirable.

We return to the question whether all other modes of treatment, if they heal, are also under the same domain of the homœopathic natural law as that of true Hahnemannian practice. In § 186 Hahnemann speaks of local diseases caused by external injuries, which, however, draw the whole organism into sympathy. With the treatment of such affections surgery is occupied. In laying down the range of this branch of therapeutics one necessary condition is enforced, which must be satisfied if the surgical interference should be a healing measure. The surgical measures must be adapted to the requirements of the case, or they must fail and amount to mere butchery. This adaptation of measures, then, proceeds just as well according to the law of healing spoken of above as the healing by appropriate medicine. Nay, inasmuch as "*the whole living organism requires active dynamic aid in order to accomplish the work of healing * * * then the business of the dynamic physician and his homœopathic aid finds its place*" (§ 186).

This shows that surgical aid is the mechanical part of the treatment, which does by no means dispense with the dynamical part, because it is the life-force in the last instance which does the healing, not the splint, or the suture, or the ligature, or the mutilation of the parts. Therefore the surgical treatment is no less under that law of the equality and contrariety of action and reaction—by which "*the external impediments to healing to be expected by the life-force alone can be annihilated mechanically*" § 186—than the internal homœopathic treatment.

Now, the modes of treatment alluded to above, as massage, water treatment, passive gymnastics, inhalation of compound oxygen, wool habiliment, pneumatic method, etc., etc., might be ranged under the head of surgical treatment, with the difference that here no external injuries are to be remedied by manual and instrumental interference, but simple manipulations and methods are applied with a view to help the life-force to resume its normal state by acting upon the various organs locally, and through them upon the various systems subordinate to the life-force. How far these numerous modes of treatment may succeed in healing is difficult to say, because it is well known that people are apt to praise a treatment they have undergone, and to boast of serious operations, even if they had not the desired effects, perhaps because it is painful to acknowledge that they went to so much expense in vain. The truth is that these local treatments cannot supplant and supersede the strict Hahnemannian treatment. They may do good when they are calculated to give the patient time and opportunity to recover from maltreatment with narcotics and wrong medicines in large doses, or afford rest and protection from disturbing circumstances. There are, no doubt, men especially endowed with a talent which others do not possess, in following up a simple local treatment of their own, and they may cure their patients by simple hygienic means and diet. All this may tend to consider such modes of treatment as modes of healing. But we have here not to pass judgment upon their merits in a therapeutical way, but to consider whether, if successful, they must be subsumed under the general homœopathic law. Like surgery they must proceed to take the appropriate measures suited for each case, and that is sufficient to claim that their success is owing to the same law which Hahnemann pronounced to be the only law of healing, viz.: *similia similibus curantur.* In the case of massage cited at the last meeting, the life-force was restored after one hour's manipulation. But a counter-statement was made that a dose of Arnica$^{cm}$ had the same effect in a similar case. This is certainly the "*cito, tuto et jucunde*" of Hahnemann, claimed by him for homœopathic healing, and the law is exemplified in the most striking manner.

The objection may be raised that if we acknowledge in the third law of motion that homœopathic natural law of which Hahnemann speaks in § 26, it is too general to be applicable to all modes of treatment, and therefore proves too much. If everything in the world and every action is subject to that law, it will, of course, also cover any mode of treatment, and there will be nothing characteristic in it to discriminate between treatment and healing. It is true also that malpractice proceeds according to this law, and since it is claimed for the general homœopathic law of nature presiding over the law of healing, there seems to be a contradiction in it which condemns it as unjustifiable. But surely the fact that malpractice as well as good practice are under the same law, it does not follow that it is exempted from its operation in good practice. Malpractice is not healing, good practice is healing. Malpractice proceeds under the law, but with the opposite effect to that of good practice. The means employed in malpractice are not adequate to the purpose, and therefore they fail. The means of good practice are adequate, and therefore they heal. This is the case in modes of treatment which are applied to the different organs of the body under the head of local treatment. As far as the medicinal treatment is concerned, where the medicines are administered so as to be taken up by the general system, the homœopathic mode of treatment is decidedly the best and safest; and there can be no doubt that it is the good practice founded upon the third law of motion which here is claimed for the law of healing as a mode of motion, not for the reverse.

This is considering the question according to primary scientific principles.

Now we might refer to a principle which for its simple common sense appeals to every sensible mind, physician or not, and it is this, popularly expressed: *An object will only be attained if the proper means are applied.*

This principle holds good in anything that man can undertake for any purpose whatever. It is applicable to the requirements of healing, whatever means may be used to secure that end.

It includes all the internal and external means by medicinal, mechanical, physical, or chemical methods. It implies in all of them the necessity of selecting and administering only those means which actually lead to healing. For there are many methods which will not effect cures, and come only under the head of treatment. Many are justified if in hopeless cases they alleviate suffering without jeopardizing life by injurious after-actions. But the healing art consists in individualizing every case and adopts the treatment that promises the required help. Then it happens that cures are wrought without administration of homœopathic potencies, as mentioned above in the case of massage. But the fact that a dose of Arnica$^{cm}$ had the same curative effect as the mechanical and mesmeric method shows that we must not concede too much. Most of the methods outside of Homœopathy are to be considered as local applications not only to the skin but also to the various organs of the body: the muscles, the joints, the bones, the rectum and intestines, the eyes, the ears, the nose, the head, the extremities, the genital organs, the respiratory tract, and almost every organ of the body has been ministered to to remove diseased states, or to act from one organ upon the other and upon the whole system.

Now, Hahnemann, knowing all this, struck a new path altogether by directing his healing efforts to the highest authority of the organism, the life-force, as the governor and regulator of all its various organs subservient only to the intellectual organs of the mind. If the proper means consisting in the potentiated remedy are selected according to symptoms, simility is applied, the life-force takes care of the necessary localization, and the diseased state in the organs is removed, because the proper means have been applied in order to convert the state of disease into one of health. From this it is clear what under homœopathic treatment the proper means of healing are.

If now, we say, the homœopathic law applies just as well to all kinds of treatment, if they actually make the sick well, as homœopathic treatment does, there can be no fanaticism in it. For that is the homœopathic law, that means, whatever they may be, are used to accomplish the end of healing. So you are not expected to heal a broken leg by a dose of Symphytum high, for only by the adaptation of the ends by means of a splint the uniting of the bones can be obtained, and this is done according to homœopathic law. But no splint will help if the bones in spite of all adaptation will not unite. Then the Symphytum comes in and the life-force is enabled to heal by the same law of adequate means, according to that same universal law of healing, the homœopathic law. Likewise the convalescence of the sick by the mere efforts of nature, called *vis medicatrix naturæ,* occurs according to that same universal homœopathic law by proper rest, nourishment, and observance of hygienic rules.

It has been said that the *similia similibus* is paramount in therapeutics. If we understand by this term the administration of medicines, this is true. If we understand by it the general treatment of the sick, which includes not only the administration of medicine, but also the management in regard to regimen, local application, and surgery, it is also true. Therapeutics means originally the general attendance to the sick, without special reference to medicine, probably because the first attendance in the olden time of Greece meant surgical treatment. It would, therefore, seem desirable to keep this general meaning of the term, and use another for the administration of remedies —as Pereira has it, Acology. We are, after all this, from the standpoint of Hahnemann, justified in calling the *similia* maxim the homœopathic law in Therapeutics and Acology. But inasmuch as all methods of restoring the disturbed integrity of the organism depends upon a homœopathic natural law, it is the universal law of healing. This, however, relates to all methods of treatment, under the indispensable condition only that they heal the sick, not only in theory, but in practice. If the practice would not bear out the theory, it would be less than the embodiment of that abominable principle: the end justifies the means.

When we analyze the law of adaptation of the means to their purpose, to which we referred as the universal principle of healing, we shall find its scientific expression also in the third law of motion. Adaptation of the means to the end is nothing else, if it comes to action, than that the means are the action which is able to produce a reaction in the body, such as to produce the desired result. The action is, no doubt, in so far similar to the reaction, as both are forces acting in contrary directions. The acting and reacting forces cannot be identical, because, even if they were of the same kind, they are as different as they can be, because opposed to each other; they are similar and contrary simultaneously. The law expressed in the formula, *similia similibus curantur*, finds its application in every system of motion by changing the copula for "æquantur," and the third law of motion is, therefore, the homœopathic natural law, of which Hahnemann speaks, in § 26.

If such reasoning is fanaticism, then there is, at least, method in the madness which recommends it, and it is nothing less than the Jesuitical principle above mentioned turned into its contrary: *the means justify the end.*

It may amuse you to turn to Walter Scott for the confirmation of the universality of the homœopathic principle, when in *Rob Roy*, chapter II, he applies it as the fundamental principle of all moral counting, the great ethic rule expressed in this way: "Let A do to B as he would have B do to him; the product will give the rule of conduct required."

A stands for the action, B for the reaction, both in contrary direction, and the result is the equalization of both on the ground of humanity.

BROOKLYN, January 21st, 1892.

